# Effects of Songs Recorded by Parents on the Vital Signs of Preterm Infants: A Randomized Controlled Trial

**DOI:** 10.3390/children12020146

**Published:** 2025-01-27

**Authors:** Yoshinori Aoki, Yuusaku Kota, Mika Shimada, Tomoko Taniguchi, Saya Yamauchi, Misaki Matsusaka, Kaoru Hamasuna, Yuuko Watanabe, Yuki Kodama, Hiroshi Moritake

**Affiliations:** 1 Division of Pediatrics, Faculty of Medicine, University of Miyazaki, 5200 Kiyotakecho Kihara, Miyazaki 889-1692, Japan; hiroshi_moritake@med.miyazaki-u.ac.jp; 2 Perinatal Center, University of Miyazaki Hospital, 5200 Kiyotakecho Kihara, Miyazaki 889-1692, Japan; yuusaku_kota@med.miyazaki-u.ac.jp (Y.K.); mika_shimada@med.miyazaki-u.ac.jp (M.S.); tomoko_taniguchi@med.miyazaki-u.ac.jp (T.T.); saya_yamauchi@med.miyazaki-u.ac.jp (S.Y.); misaki_kamimura@med.miyazaki-u.ac.jp (M.M.); kaoru_hamasuna@med.miyazaki-u.ac.jp (K.H.); yuuko_ozono@med.miyazaki-u.ac.jp (Y.W.); yuki_kodama@med.miyazaki-u.ac.jp (Y.K.); 3 Obstetrics & Gynecology, Faculty of Medicine, University of Miyazaki, 5200 Kiyotakecho Kihara, Miyazaki 889-1692, Japan

**Keywords:** preterm, prematurity, music, recorded parental song, respiration

## Abstract

**Background:** Preterm infants often have unstable vital signs and prolonged hospital stays that can hinder parent–infant bonding, especially under COVID-19 restrictions. This study aimed to evaluate whether listening to songs recorded by parents was effective in stabilizing the condition of premature infants. **Methods:** This randomized controlled study was conducted at the University of Miyazaki Hospital from October 2022 to March 2024 during the COVID-19 pandemic period. The participants were preterm infants born at less than 33 weeks gestation and their parents, all of whom recorded songs. The recorded songs were played daily to the infants in the intervention group, while the control group received usual care. Primary outcomes included vital signs (respiratory rate, pulse oximetry saturation, heart rate) and activity level. **Results:** Data for 33 preterm infants (intervention, *n* = 17 [total 749 sessions]; control, *n* = 16 [total 721 sessions]) were analyzed for changes in vital signs and activity levels. The intervention reduced infants’ respiratory rates (4.1 [95% CI: 2.5–5.6], *p* < 0.001) and slightly but statistically significantly increased pulse oximetry saturation (0.6 [95% CI: 0.02–1.2], *p* < 0.044). **Conclusions:** Recorded parental songs were found to safely stabilize the respiratory status of preterm infants and may serve as an accessible intervention to support parent–infant attachment, particularly in settings with restricted parental visitation.

## 1. Introduction

Prematurity is the leading cause of neonatal mortality and is associated with adverse outcomes [1,2]. Worldwide, an estimated 13 million babies are born preterm each year, accounting for approximately 10% of all births [3]. While advances in medical care have improved the survival rate of preterm infants [4], extremely preterm infants requiring invasive intensive care often have unstable respiratory and circulatory status, leading to frequent fluctuations in their vital signs. Additionally, these infants may remain hospitalized for several months, contributing to significant psychological distress for the parents and impeding parent–infant attachment [5]. In recent years, developmental care and family-centered care have been increasingly adopted, with the aim of not only ensuring survival but also promoting development and enhancing parent–infant bonding [6,7,8]. Such active parental involvement supports attachment formation and contributes to stabilizing the condition of preterm infants. However, due to the widespread outbreak of COVID-19, many facilities in Japan prohibited or strictly restricted visitation, depriving parents of opportunities to engage in the care of their infants [9,10].

Music therapy and auditory stimulation, which involve providing live music or recorded music and sounds to infants by music therapists, healthcare professionals, or family members, have been reported to stabilize the condition of preterm infants and reduce maternal stress [11,12,13]. While these interventions show promise, some studies suggest that a quiet environment may be more beneficial for preterm infants [14]. Moreover, the long-term impact of music therapy and auditory stimulation on the development of preterm infants has been minimally explored, with limited evidence available, leaving many aspects of the effectiveness of music interventions in preterm infants unclear [15,16].

In this study, parents with restricted visiting access recorded songs at home to be played for their infants. The aim was to evaluate whether listening to these songs recorded by parents was effective in stabilizing the condition of premature infants.

## 2. Materials and Methods

### 2.1. Study Design

This randomized controlled study was conducted at the University of Miyazaki Hospital, a tertiary neonatal center in Miyazaki, Japan, between October 2022 and March 2024. This period corresponds to the COVID-19 pandemic and its aftermath, during which visitation was prohibited or strictly restricted in most hospitals across Japan. The hospital was chosen because it serves as a regional center for preterm infants, the target population of this study, making it an ideal setting for recruitment. The study protocol and analysis plan were registered (UMIN000049413).

### 2.2. Ethical Considerations

This study was conducted in accordance with the Declaration of Helsinki. Ethical approval was obtained from the ethics committee of the University of Miyazaki Hospital (I-0063). Written informed consent was obtained from the parents who agreed to participate in this study.

### 2.3. Study Population

Infants younger than 33 weeks and 0 days of gestation who were admitted to the hospital before 7 days of age or at a postmenstrual age of 28 weeks and 0 days were eligible for enrollment. Infants were excluded if they had (1) major congenital anomalies and (2) neurological sequelae before randomization (e.g., intraventricular hemorrhage, periventricular leukomalacia, or seizures).

The sample size was calculated based on the study’s primary outcomes, initially considering the results of developmental testing at 18 months of corrected age. A 10% difference between the two groups was expected for the developmental testing, with a standard deviation of 13 estimated from previous studies [17,18]. It was calculated that a total of 60 infants should be included, with a 5% margin of error, 80% power value, and 15% loss to follow-up. However, enrollment was terminated at 34 participants, before reaching the initial planned sample size, because family visitation restrictions were lifted as the COVID-19 pandemic subsided. Therefore, we decided to adopt the effects of the recorded parental songs on the vital signs and activity levels of the infants as the primary outcomes.

### 2.4. Randomization and Blinding

This study followed the CONSORT guidelines [19,20]. The participants were assigned in a 1:1 ratio and stratified by gestational age (gestational age < 28 weeks or ≥28 weeks). The randomization list was generated by a researcher who was not otherwise involved in the study, using a computer program to generate random numbers. The parents and the outcome examiners were blinded to the group allocations.

### 2.5. Intervention

The parents of all participants recorded their own voices singing songs of their choice, such as lullabies or their favorite songs, on a voice recorder. The actual instructions for parents and examples of songs are shown in the Supplementary Table (Supplementary Materials, Table S1). For participants in the intervention group, the intervention was initiated after 7 days of age and 28 weeks of postmenstrual age based on a previous study that evaluated auditory development in preterm infants [21]. The infants listened to the recorded songs at a fixed time for 30 min every day, based on previous studies [15,22,23]. In accordance with previous studies and recommendations from the American Academy of Pediatrics, the voice recorder was placed 20 cm away from the ears, and the volume was adjusted to 40 decibels [15,24]. The intervention continued until the participants reached a postmenstrual age of 37 weeks and 0 days. While previous studies often involved interventions lasting only a few days [22,23], studies evaluating long-term effects have extended the intervention period over several weeks [15]. Therefore, in this study, the intervention was continued until the participants reached 37 weeks and 0 days. For participants assigned to the control group, the usual care was provided while monitoring noise levels to ensure appropriate noise control.

### 2.6. Outcomes

The primary outcomes were the infants’ vital signs (respiratory rate [RR], pulse oximetry saturation [SpO_2_], and heart rate [HR]) and activity level. The activity level was rated from 1 (deep sleep) to 6 (crying) [25]. Vital signs were extracted from the continuous monitoring system immediately before and at the end of the intervention. Additionally, the activity level was recorded by the caregiver. The vital signs and activity levels of the control group were recorded at the same time as the intervention group. In addition, a questionnaire on parent–child attachment (the Japanese version of the Maternal Attachment Inventory) was completed at the time of discharge [26,27].

The safety of this intervention was investigated based on the occurrence of severe hearing impairment.

### 2.7. Statistical Analysis

Data are shown as mean ± standard deviation for normally distributed continuous variables, as median and interquartile range (IQR) for other continuous variables, and as number and percentage of the total number of infants for categorical variables. Statistical analyses were performed using SPSS Statistics (ver. 28; IBM Corp., Armonk, NY, USA). Differences between the groups were evaluated using Student’s *t*-test or the Mann–Whitney U test for continuous variables and Fisher’s exact test for categorical variables. A *p*-value of <0.05 was considered statistically significant.

## 3. Results

**Figure 1:**
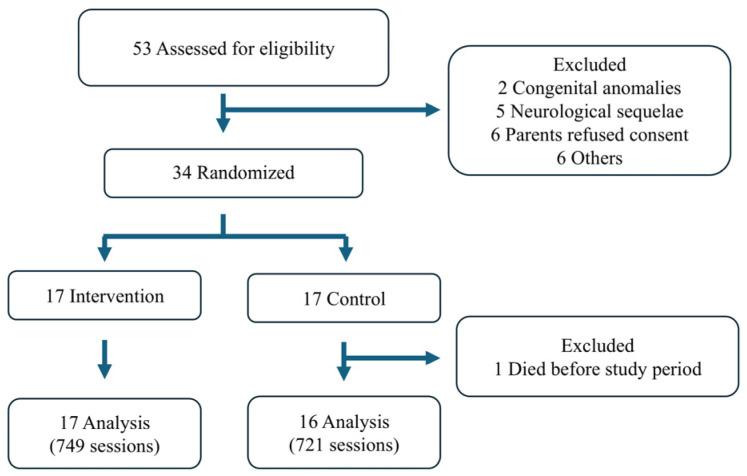
Study flowchart.

A total of 53 eligible infants were admitted to the hospital during the study period. Of these, 19 infants were excluded from the study. Two had congenital anomalies, five had neurological sequelae, six lacked parental consent, and six were excluded for other reasons, such as maternal mental illness. Consequently, 34 patients were enrolled and randomized. Of the 34 infants, 17 were allocated to the intervention group and 17 were allocated to the control (usual care) group. Due to the death of one patient prior to the study period, the analysis was conducted on the remaining 33 patients (17 in the intervention group and 16 in the control group) (Figure 1).

The baseline characteristics of the infants are described in Table 1. The gestational age, birth weight, sex, number of multiple births, mode of delivery, Apgar scores, and number of patients who underwent endotracheal intubation were not significantly different in the two groups.

**Figure 2:**
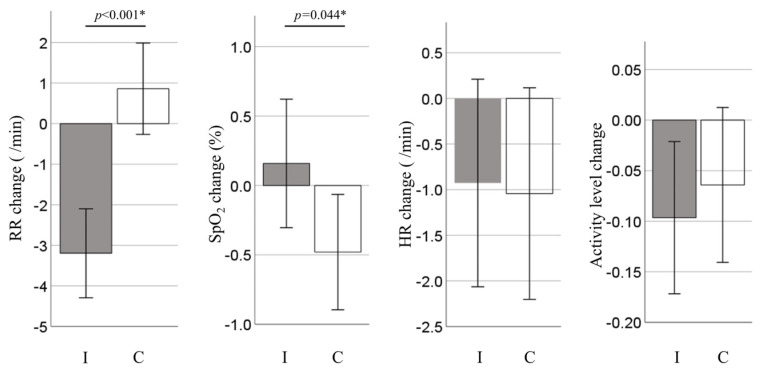
Changes in vital signs and activity levels from before to after the intervention. Bars indicate mean values ± SD. RR, respiratory rate; SpO_2_, pulse oximetry saturation; HR, heart rate; I, intervention group; C, control group. * Student’s *t*-test.

Table 2 shows the respiratory rate (RR), pulse oximetry saturation (SpO_2_), heart rate (HR), and activity levels of the infants before and immediately after intervention, as well as the changes in these values in the two groups. Compared with the control group, the RR in the intervention group was significantly decreased (*p* < 0.001, 4.1 [95% CI: 2.5–5.6], Table 2 and Figure 2). The increase in SpO_2_ in the intervention group was slight but significantly higher than that in the control group (*p* = 0.044, 0.6 [95% CI: 0.02–1.2]; Table 2 and Figure 2). As for the HR and activity levels, no significant differences were observed between the two groups. Parent–child attachment was assessed using the Japanese version of the Maternal Attachment Inventory, with a mean (±SD) score of 99.2 (±5.9) out of 104.

In terms of safety, one patient in the intervention group was diagnosed with severe bilateral hearing loss. The diagnosis was bilateral cochlear nerve aplasia based on head MRI findings; hence, the hearing loss in this infant was considered to be congenital and unrelated to the intervention in this study.

## 4. Discussion

In this study, we played recordings of parents singing to hospitalized preterm infants. The respiratory rate decreased, while the pulse oximetry saturation showed a slight but statistically significant increase. We believe this indicates that recorded parental songs help stabilize the respiratory status of preterm infants. In a previous study and systematic review, there were no changes in respiratory rate [11,28], but another meta-analysis showed a decrease in respiratory rate [12]. However, these studies included different interventions, which makes it difficult to draw conclusions. This present study suggests that recorded parental songs may help stabilize the respiratory status of preterm infants.

The term ‘music therapy’ generally refers to the interventions provided by certified professional music therapists [13], which can bring physiological and psychological stability to neonates. While the songs recorded by parents in this study may lack the musical quality of the live music provided by professional music therapists, previous studies have shown that neonates can distinguish between their mother’s voice and the voices of others [29,30]. Moreover, a mother’s voice has been found to more effectively enhance the development of networks in brain regions associated with language development [29]. Although research on fathers’ voices is limited [31], hearing both parents’ voices is beneficial for neonates. Furthermore, for parents who cannot visit, the aspect of being involved in some way is important. Despite restrictions on parental visitation, the attachment-related questionnaire scores were comparable to those reported in previous studies [27,32]. Participation in this study may have contributed to the development of parental attachment.

Apart from pandemics, visitation can be restricted due to parental illness, geographical issues, or socioeconomic challenges. This study indicates that recorded parental songs may help stabilize the respiratory status of preterm infants. The advantage of this method is that it can be implemented with simple devices. We believe it can be applied in regions with limited medical resources or computer networks.

In terms of safety, one participant in the intervention group was diagnosed with severe bilateral hearing loss, which was determined to be due to bilateral cochlear nerve aplasia based on head MRI findings; therefore, the hearing loss in this infant was considered to be congenital and unrelated to the intervention of this study. Previous studies have rarely reported adverse events associated with music therapy [28]. We believe that music therapy is safe as long as attention is paid to sound levels.

This study included extremely preterm infants (born before 28 weeks of gestation) relative to previous studies [12,13]. The intervention began at a corrected gestational age of 28 weeks, in line with previous ABR studies [21]. The results suggest that recorded parental songs may also be effective for such extremely preterm infants.

This study had several limitations. First, it was a single-center study. Second, while both parents and evaluators were blinded, the caregivers were not. Additionally, the study concluded with a smaller sample size than originally planned. However, we were able to observe statistically significant changes in the infants’ vital signs. The long-term effect of this intervention on neonates remains unknown and will be evaluated in future studies.

## 5. Conclusions

This study demonstrated that listening to recorded songs sung by parents significantly stabilized the respiratory status of preterm infants by reducing their respiratory rates and slightly increasing their pulse oximetry saturation. These findings highlight the potential of recorded parental songs as an accessible and cost-effective intervention for preterm infants, particularly in contexts where parental visitation is restricted due to pandemics and geographic or socioeconomic challenges. While the intervention was shown to be safe and effective, further studies are needed to confirm these findings and evaluate the long-term developmental effects of recorded parental songs on preterm infants.

## Figures and Tables

**Table 1:** Baseline maternal and infant characteristics according to treatment group.

	Intervention (*n* = 17)	Control (*n* = 16)	*p*
Gestational age (weeks), median (IQR)	28 (25–29)	28.5 (27–29)	0.29 *
Birth weight (g), median (IQR)	787 (629–1005)	1077 (741–1220)	0.22 *
Sex (male), *n* (%)	7 (41)	9 (56)	0.49 **
Multiple births, *n* (%)	4 (24)	6 (38)	0.47 **
Cesarean section delivery, *n* (%)	14 (82)	13 (81)	1 **
Apgar 1 min, median (IQR)	5.5 (4–7)	5.5 (4.8–6.3)	0.44 *
Apgar 5 min, median (IQR)	8 (7–8.3)	7 (6.8–8)	0.82 *
Intubation at birth, *n* (%)	12 (71)	12 (75)	1 **

IQR, interquartile range. * Mann–Whitney U test. ** Fisher’s exact test.

**Table 2:** Effect of intervention on vital signs and activity levels.

	Intervention	Control	*p*
Respiratory rate (/min)			
Before	49.8 ± 12.4	48.7 ± 10.6	
End	46.6 ± 12.1	49.5 ± 11.1	
Change	−3.2 ± 13.9	0.9 ± 13.9	<0.001 *
Pulse oximetry saturation (%)			
Before	95.2 ± 4.7	95.6 ± 4.4	
End	95.5 ± 4.9	95.1 ± 6.0	
Change	0.2 ± 6.5	−0.5 ± 5.7	0.044 *
Heart rate (bpm)			
Before	155 ± 12	157 ± 13	
End	155 ± 13	156 ± 14	
Change	−0.9 ± 15	−1.0 ± 15	0.117 *
Activity level (1–6)			
Before	1.50 ± 0.79	1.48 ± 0.80	
End	1.41 ± 0.79	1.43 ± 0.85	
Change	−0.10 ± 1.0	−0.06 ± 1.0	0.554 **

Values are expressed as the mean ± standard deviation. “Before” means the measurement taken immediately before the intervention. “End” means the measurement taken at the end of the intervention. “Change” refers to the difference between “End” and “Before”. Specifically, for each variable, the “Change” value was calculated by subtracting the “Before” value from the “End” value. * Student’s *t*-test. ** Mann–Whitney U test.

## Data Availability

The data presented in this study are available on request from the corresponding author.

## Supplementary Materials

The following supporting information can be downloaded at: https://www.mdpi.com/article/10.3390/children12020146/s1, Table S1: Guidelines for Recordings and Examples of Songs.

## Abbreviations

The following abbreviations are used in this manuscript:

RR	Respiratory rate
SpO_2_	pulse oximetry saturation
HR	Heart rate
